# 5-Channel Polymer/Silica Hybrid Arrayed Waveguide Grating

**DOI:** 10.3390/polym12030629

**Published:** 2020-03-10

**Authors:** Sheng-Rui Zhang, Yue-Xin Yin, Zi-Yue Lv, Ding-Shan Gao, Xi-Bin Wang

**Affiliations:** 1 Wuhan National Laboratory for Optoelectronics and School of Optical and Electronic Information, Huazhong University of Science and Technology, Wuhan 430074, China; u201713991@hust.edu.cn (S.-R.Z.); 15827605011@163.com (Z.-Y.L.); 2 State Key Laboratory of Integrated Optoelectronics, College of Electronic Science and Engineering, Jilin University, Changchun 130012, China; yinyx18@mails.jlu.edu.cn

**Keywords:** integrated optics, polymer optical waveguides, optical communication systems, planar lightwave circuit

## Abstract

A 5-channel polymer/silica hybrid arrayed waveguide grating (AWG), fabricated through a simple and low-cost microfabrication process is proposed, which covers the entire O-band (1260–1360 nm) of the optical communication wavelength system. According to the simulation results, the insertion loss is lower than 4.7 dB and the crosstalk within 3-dB bandwidth is lower than ~−28 dB. The actual fiber–fiber insertion loss is lower than 14.0 dB, and the crosstalk of the 5 channels is less than −13.0 dB. The demonstrated AWG is ideally suitable for optical communications, but also has potential in the multi-channel sensors.

## 1. Introduction

With the rapid growth of data center interconnection, a coarse wavelength division multiplexing (CWDM) system in the O-band (1260–1360 nm) becomes the first choice for metro-access networks, owing to its low cost in equipment, operation, and maintenance [1,2]. To realize wavelength division, many kinds of structures have been proposed, including arrayed waveguide grating (AWG), planar concave grating (PCG) [3], and microring resonator (MRR) [4]. Specifically, AWG has advantages of low cost, large fabrication tolerance, and high performance, and has been widely studied. As the centerpiece of wavelength division multiplexing (WDM), AWG has been fabricated by silicon-on-insulator [5], SiO_2_ [6,7], and polymers [8,9,10,11]. Though AWG has been highly developed by the standard industry technology of planar lightwave circuits (PLCs), and the cost is being gradually reduced by the mature fabrication process of silica-based waveguide [12,13,14], the polymer AWG has its own advantages and can avoid complex preparation processes such as thermal oxidation, plasma-enhanced chemical vapor deposition (PECVD), and inductively coupled plasma (ICP) etching [12,13,14]. Compared with silica-based devices, polymer-based devices can skip several steps in manufacture, resulting in reduced prices for the setup and, especially for small batch sizes, a reduced price per unit [15,16,17]. Moreover, due to good compatibility on diverse substrates, polymer-based devices can provide suitable platforms for the hybrid integration of photonic chips [18,19,20]. Besides having the advantages of low cost, small birefringence, easy control of refractive index, large thermo-optic coefficients, and easy fabrication, compared with inorganic materials, biocompatibility is an especially important and unique feature of polymers, which makes them an ideal material platform for photonic integrated waveguide biosensors [18]. We built up a multi-channel sensor system based on separate devices, including a dense wavelength division multiplexing (DWDM) and sensors [21]. Furthermore, CWDM can supply larger bandwidth compared with DWDM, which is much more suitable to build up a multi-channel sensor system and fabricate monolithic multi-channel sensors [22]. There is plenty of research about CWDM [23,24], however, less of it is based on polymer materials [25,26], to the best of our knowledge. Still, it remains challenging to reduce the insertion loss and the crosstalk of the polymeric AWG. 

In this paper, we designed and fabricated a 5-channel AWG based on polymer/silica hybrid waveguide, covering the entire O-band of the optical communication wavelength system. According to the simulation results, the insertion loss is lower than 4.7 dB in the O-band, and the crosstalk within 3-dB bandwidth is lower than ~−28 dB. Also, the AWG was successfully fabricated through a microfabrication process. Over the O-band, the measured fiber–fiber insertion loss is lower than 14.0 dB. Within 3-dB bandwidth, the crosstalk of each channel is lower than –13.0 dB. The proposed AWG is well-suited for large-scale manufacture, and it has various potential applications, like hybrid integration optical circuits and the monolithic multi-channel sensors. In the future, a packaging method will be developed to improve the performance of the AWG.

## 2. Device Design and Simulation

In our design, we chose SU-8 2002 photoresist as core material, polymethy-methacrylate (PMMA) as cladding, and silica as substrate, whose refractive indexes are 1.571, 1.483, and 1.446, respectively, measured with an M-2000UI variable angle incidence spectroscopic ellipsometer at 1550 nm wavelength (J.A.Woollam Co., Inc., Lincoln, NE, USA). SU-8 2002 photoresist is a commercially available negative UV photoresist, which has very high transmission for wavelengths above 400 nm [27] and exhibits relatively good chemical and thermal stability (with a glass transition temperature of Tg ≈ 200 ℃ and a degradation temperature of Td ≈ 380 ℃) [28]. Here, we chose silica as the substrate for the following results. Firstly, polymer and silica materials are immiscible. Secondly, the mechanical strength of silica is much greater than polymer materials, which will improve the yield and reliability of the ICP etching, providing a possibility for hybrid integration for polymer and silica. Thirdly, the coefficient of linear thermal expansion of silica is much smaller than that of the polymer and silicon. They are 0.5 × 10^−6^, 0.55 × 10^−4^, and 2.5 × 10^−6^ K^−1^, respectively [20].

**Figure 1:**
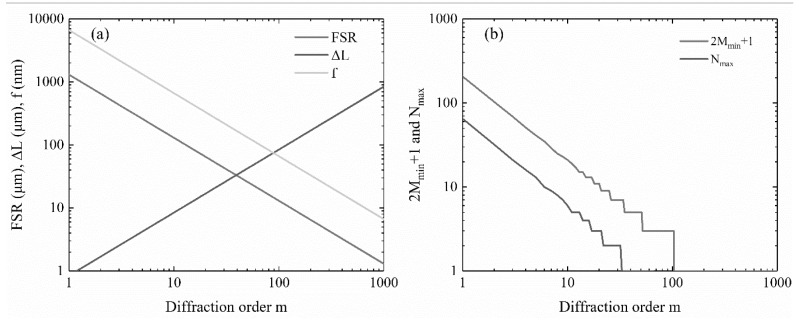
(**a**) Relations between the diffraction order *m* and the length difference of adjacent arrayed waveguides Δ*L*, focal length of slabs *f,* and FSR. (**b**) Relations between the diffraction order *m* and the maximum number of I/O channels Nmax and the minimum number of arrayed waveguides 2*M*_min_ + 1.

The design of the waveguide dimensions and AWG geometry was performed with commercial software WDM Phasar (Optiwave Corporation, Ottawa, ON, Canada) under consideration of the refractive indices of the applied materials. The waveguide dimensions were chosen to be as small as possible, while still being reliably producible with conventional contact lithography. Ultimately, this led to a few-mode behavior of the produced AWG due to size limitations, and certain mode dispersion. Nevertheless, we applied mode-division multiplexing in the end of output waveguides to decrease the loss caused by mode dispersion. What is more, it will increase the channel number. The length differences of adjacent arrayed waveguides, focal length of slabs, and FSR are given by [29].

(1) $$\Delta L=\frac{m\lambda_{0}}{n_{c}}$$

(2) $$f=\frac{n_{s}d^{2}}{m\Delta \lambda }\frac{n_{c}}{n_{g}}$$

(3) $$FSR=\frac{\lambda_{0}}{m}\frac{n_{c}}{n_{g}}$$

where *λ*_0_ is the wavelength in free space, *d* is the pitch of adjacent waveguides, *n*_c_ and *n*_s_ are effective refractive indexes of the rectangular waveguide and slab waveguide, respectively, and *n*_g_ is the group refractive index. The diffraction order *m* is an important parameter. Once the diffraction order was determined, some geometries of the device were also determined. Figure 1a shows the relations between the diffraction order *m* and the length difference Δ*L*, focal length f, and FSR. Figure 1b shows the relation between the diffraction order *m* and the maximum number of I/O channels *N*_max_ and the minimum number of arrayed waveguides 2*M*_min_ + 1. In this paper, the number of I/O channels was determined to be 2*N* + 1 = 5, therefore, the diffraction order should be taken as *m* = 11. The minimum number of array waveguides was 2*M*_min_ + 1 = 19. In fact, the number of arrayed waveguides should be taken as large as possible in order to decrease the loss. However, it is also constrained by the device size. Finally, we chose 2*M* + 1 = 25. In summary, the optimized parameter values of the AWG multiplexer are listed in Table 1.

**Figure 2:**
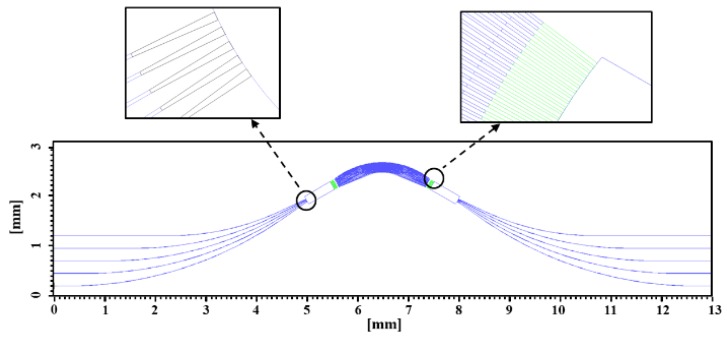
Layout schematic of the fabricated AWG device.

As shown in Figure 2, the entire footprint of the AWG layout was 13000 × 2463 μm^2^, and the footprint of the array waveguide region was 3065 × 825 μm^2^. At the ends of input and output waveguides, we used taper waveguide structure to reduce the coupler loss. The width of taper waveguides changed from 3 to 7 μm linearly. The length of taper structure was 75 μm. Also we used taper waveguides between slab waveguides region and array waveguides region. Limited by the number of array waveguides, the width of waveguides changed from 4 to 3 μm linearly. The lengths of taper waveguides were set at 70 μm. 

**Figure 3:**
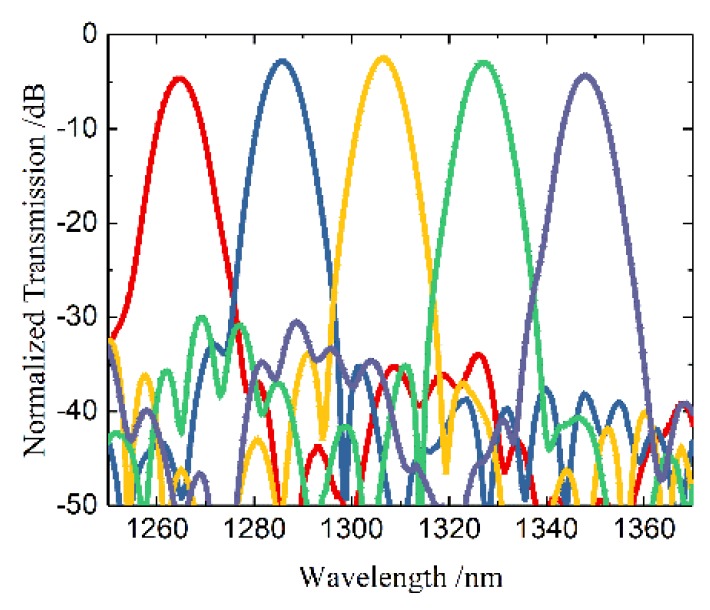
The simulated transmission spectrum of the AWG.

The simulated transmission spectrum is illustrated in Figure 3. As shown in Figure 2, the central wavelengths of each channel were 1264.5, 1285.6, 1306.7 nm, 1327.2, and 1347.7 nm, respectively. The insertion loss was lower than 4.7 dB in the O-band. 3-dB bandwidth of each channel was 8 nm. The crosstalk within 3-dB bandwidth was lower than ~−28 dB.

## 3. Device Fabrication and Characterization

**Figure 4:**
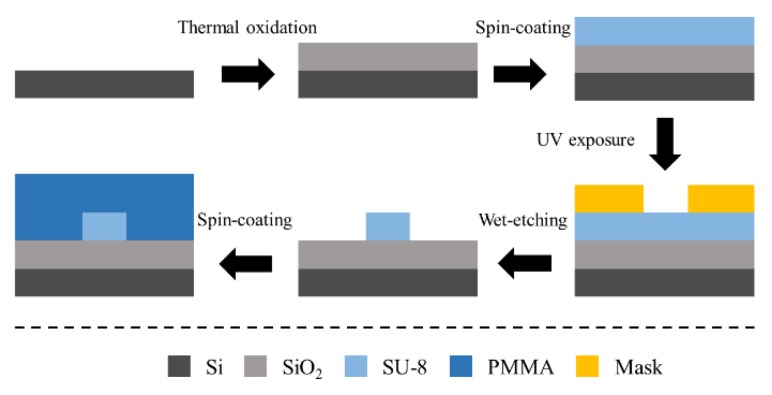
The fabrication process of the AWG.

**Figure 5:**
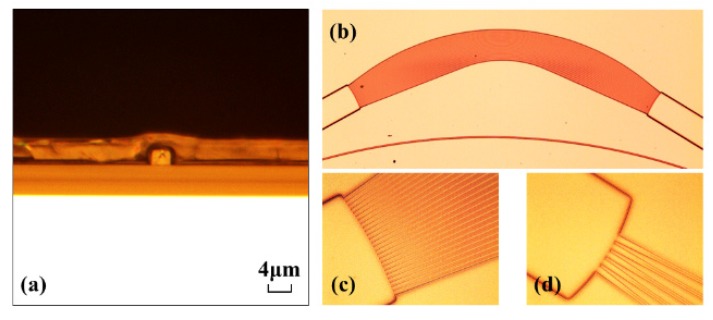
The micrographs of the fabricated AWG: (**a**) the crossing diagram, (**b**) the overall structure diagram and (**c,d**) partial enlarged diagram of the fabricated AWG.

The fabrication process flow of the AWG is shown in Figure 4. Negative photoresist SU-8 2002 was spin-coated as the core layer onto the silica substrate layer at a rotational speed of 1500 r/min and prebaked at 95 ℃ for 10 min. Then the coated chip was exposed to UV light from a mercury discharge lamp (peak emission wavelength, 365 nm; irradiance, 23 mW/cm^2^_,_ ABM-USA, Inc., San Jose, CA, USA) for 3 s and post-baked at 95 ℃ for 10 min. After that, we removed the unexposed SU-8 polymer and then hard-baked it at 125 ℃. Finally, 5-μm-thick PMMA upper cladding was spin-coated on the waveguide cores and baked at 125 ℃ for 2.5 h. The micrographs of the fabricated AWG are shown in Figure 5. Figure 5a shows the cross-section of the fabricated AWG. Figure 5b shows the overall structure diagram of the AWG. Figure 5c,d is the enlarged diagrams of the connection parts between the planar waveguide region and array waveguide region, and the connection parts between the planar waveguide region and output waveguide region, respectively. Here, the minimum distance between array waveguides was 2 μm.

**Figure 6:**
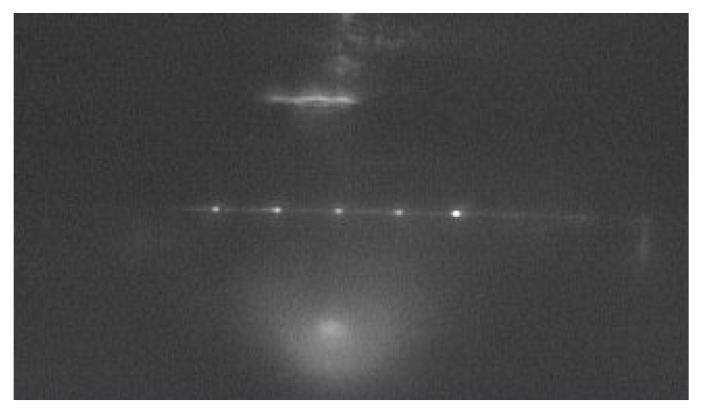
Near-field guided-mode patterns of the AWG.

**Figure 7:**
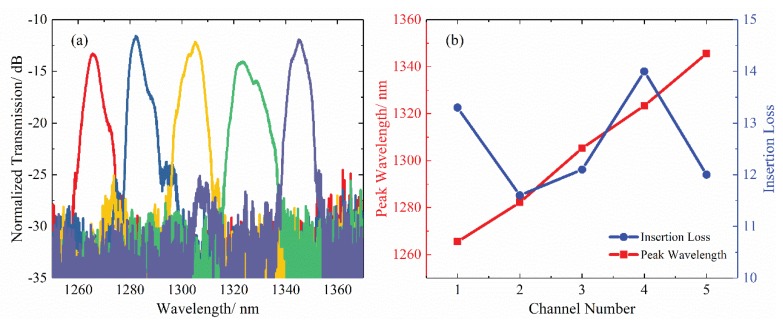
(**a**) The measured transmission spectrum of the AWG; (**b**) The peak wavelength and insertion loss of 5 channels.

The device under test (DUT) consisted of a broad-spectrum laser (SC-5, Yangtze Soton Laser, Wuhan, China) and an optical spectrum analyzer (MS9740A, Anritsu, Kanagawa, Japan). The near-field pattern of the AWG device is shown in Figure 6. The light from the broad-spectrum laser was coupled into the AWG by a lensed fiber. We monitored the transmission spectrum of each channel of the AWG with the optical spectrum analyzer. The measured transmission spectrum of the AWG is shown in Figure 6, which was basically consistent with the simulation results. From Figure 7, the peak wavelengths of each channel were 1265.5, 1282.2, 1305.3, 1323.4, 1345.6 nm, respectively. The averaged channel spacing was ~20 nm. Compared with the simulation spectrum, the peak wavelength shifting were +1, −3.4, +1.4, +3.8, and +2.1 nm, respectively.

The insertion loss of the straight reference waveguide was measured to be ~11.9 dB. The sample had a total length of 1.3 cm. The propagation loss in the reference waveguide, measured by the cutback method, was ~1.4 dB/cm. Therefore, the edge coupling loss between the waveguide and fiber was ~5 dB/facet [15]. Based on the above analysis, we can conclude that the insertion loss of the device is mainly caused by the edge coupling loss. It can be further improved by polishing the facets of the device or introducing grating coupler. The 3-dB bandwidths of each channel were 5.7, 5.3, 7.2, 10.9, and 7.0 nm, respectively. Within 3-dB bandwidth, the crosstalk of each channel was lower than −13.0 dB.

## 4. Conclusions 

In summary, a 5-channel polymer/silica hybrid AWG was designed and experimentally demonstrated. The introduction of a silica bottom layer decreased the loss and crosstalk. Besides, this fabricated process was compatible with PLC flow. The hybrid integrated devices will further improve device performance. According to the simulation results, the central wavelengths of each channel were 1264.5, 1285.6, 1306.7, 1327.2, and 1347.7 nm, respectively. The insertion loss was lower than 4.7 dB in the O-band. The crosstalk within 3-dB bandwidth was lower than ~-28 dB. Also, the AWG was fabricated successfully through a microfabrication process. The central wavelengths of each channel were 1265.5, 1282.2, 1305.3, 1323.4, and 1345.6 nm, respectively. Over the O-band, the insertion loss was lower than 14.0 dB, which mainly comes from the coupling loss between the input/output waveguide and the fibers. Within 3-dB bandwidth, the crosstalk of each channel was lower than -13.0 dB. The AWG is very well-suited for large-scale manufacturing and has broad potential applications, especially for multi-channel sensors. In the future work, the packaging method will be developed to improve the performance of the AWG.

## Figures and Tables

**Table 1:** Design parameters of 5 × 5 arrayed waveguide grating (AWG).

Number of Channels	5
Central Wavelength (nm)	1311
Channel Spacing (nm)	20
Core Size (μm^2^)	3×3
Free Spectral Range (nm)	118.18
Diffraction Order	11
Orientation Angle (deg)	30
Spacing of Arrayed and Input/Output Waveguides (μm)	9.25
Path Length Difference ∆L (μm)	9.30
Number of Arrayed Waveguide	25
